# Testosterone and Adipokines are Determinants of Physical Performance, Strength, and Aerobic Fitness in Frail, Obese, Older Adults

**DOI:** 10.1155/2014/507395

**Published:** 2014-07-02

**Authors:** Lina E. Aguirre, Irum Zeb Jan, Kenneth Fowler, Debra L. Waters, Dennis T. Villareal, Reina Armamento-Villareal

**Affiliations:** ^1^New Mexico VA Health Care System, 1501 San Pedro SE, Albuquerque, NM 87108, USA; ^2^Biomedical Research Institute of New Mexico, Albuquerque, NM 87108, USA; ^3^University of Otago, Dunedin 9016, New Zealand; ^4^University of New Mexico School of Medicine, Albuquerque, NM 87131, USA

## Abstract

In this study, we evaluated the independent and combined effects of baseline circulating gonadal, anabolic hormones and adipokines on physical function in 107 frail, obese (BMI ≥ 30 kg/m^2^), and older (≥65 yr) subjects. Our results showed significant positive correlations between circulating testosterone and insulin growth factor-1 (IGF-1) with knee flexion, knee extension, one-repetition maximum (1-RM), and peak oxygen consumption (VO_2_ peak), while no correlation was observed with estradiol. Among the adipokines, high sensitivity C-reactive protein (Hs-CRP) and leptin negatively correlated with the modified physical performance testing (PPT), knee flexion, knee extension, 1-RM, and VO_2_ peak. Interleukin-6 ( Il-6) negatively correlated with knee flexion and VO_2_ peak and soluble tumor necrosis factors receptor-1 (sTNFr1) correlated with PPT, 1-RM, and VO_2_ peak. Adiponectin correlated negatively with 1-RM. Multiple regression analysis revealed that, for PPT, sTNFr1 was the only independent predictor. Independent predictors included adiponectin, leptin, and testosterone for knee flexion; leptin and testosterone for knee extension; adiponectin, leptin, and testosterone for 1-RM; and IGF-1, IL-6, leptin, and testosterone for VO_2_ peak. In conclusion, in frail obese older adults, circulating levels of testosterone, adiponectin, and leptin appear to be important predictors of physical strength and fitness, while inflammation appears to be a major determinant of physical frailty.

## 1. Introduction

A high body mass index (BMI) is associated with impairment in activities of daily living, limitations in mobility, decreased physical performance, and increased risk for functional decline [1] leading to increased nursing home admissions [2]. The elderly obese are especially susceptible to the adverse effects of excess body fat on physical function, because of (1) decreased muscle mass and strength which occur with aging and (2) the need to carry greater weight due to obesity [3]. Investigators from our group first reported that 96% of community-living older adults with BMIs > 30 kg/m^2^ were frail, as determined by physical performance test scores, peak oxygen consumption (VO_2_  peak), and ability to perform activities of daily living [3].

Aging is associated with the decline in gonadal and anabolic hormones and increased in inflammatory cytokines. These changes are associated with loss of muscle mass and decline in strength and physical function. An increase in lean body mass, strength, and physical function has been demonstrated with testosterone [4, 5] and growth hormone (GH) [6] treatment among patients deficient of these hormones. On the other hand, aging is associated with increase in inflammatory cytokines, and high levels of these cytokines promote muscle catabolism and reduced muscle volume [7]. Since loss of muscle volume leads to reduced muscle strength and function, it is possible that the age-related hormonal decline and increased inflammation contribute to deterioration in physical function leading to frailty. Furthermore, in the obese elderly patient, the added increase in inflammatory cytokine production from the excess adipose tissues would enhance inflammation perhaps leading to worse frailty syndrome than nonobese elderly patients. However, little information is available on the contribution of the interaction of circulating hormones and adipokines (some of them proinflammatory) on frailty and physical disability in obese elderly adults.

The objective of this study was to evaluate the independent and combined effects of circulating gonadal (testosterone and estradiol) and anabolic hormones (growth hormone/IGF-1) and adipose-tissue derived factors (adipokines) on physical function, strength and physical fitness in frail obese older adults.

## 2. Methods

### 2.1. Study Design and Study Population

This study is a cross-sectional analysis of baseline data from subjects who volunteered to participate in a previous lifestyle therapy trial of sedentary, frail, and elderly obese adults [8, 9]. This study was conducted at Washington University School of Medicine in accordance with the guidelines in the Declaration of Helsinki for the ethical treatment of human subjects. The protocol was approved by the Washington University Institutional Review Board and a written informed consent was obtained from each subject. Participants were recruited through newspaper and radio advertisements. Inclusion/exclusion criteria were as reported previously [8, 9]. Briefly, participants were ≥65 years of age, with BMI ≥ 30 kg/m^2^, had sedentary lifestyle, (did not participate in regular exercise more than twice a week), had stable body weight (±2 kg) over the past year, and were on stable medications for 6 months before enrollment. Participants who were treated with bone-acting drugs (e.g., bisphosphonates, glucocorticoids, and sex-steroid compounds) during the previous year were excluded from participation. At enrollment, these subjects should not have cardiorespiratory or neuromuscular diseases that would limit their ability to exercise, diabetes mellitus, osteoporosis, hyperparathyroidism, chronic liver disease, uncontrolled or untreated hyperthyroidism, or significant renal impairment.

### 2.2. Physical Function

Physical function to determine frailty was assessed using the modified physical performance test (PPT) as previously described [8, 9]. The modified PPT includes seven standardized tasks (walking 50 ft, putting on and removing a coat, picking up a penny, standing up from a chair, lifting a book, climbing one flight of stairs, and performing a progressive Romberg test) plus two additional tasks (climbing up and down four flights of stairs and performing a 360-degree turn). The score for each task ranges from 0 to 4; a perfect score is 36.

### 2.3. Muscle Strength Testing

#### 2.3.1. Knee Flexion and Knee Extension

Isokinetic knee extensor and flexor strength were evaluated by using a Biodex System 3 dynamometer (Shirley, NY) as previously described [10]. During the testing, the participants were seated with their backs supported and hips positioned at 120° of flexion and secured to the seat of the dynamometer with thigh and pelvic straps. All tests were performed on the right leg. Testing was performed at an angular velocity of 60° per second. The best of the 3 maximal voluntary efforts for each of the knee flexion and extension was used as the measure of absolute strength and reported as peak torque at 60° in Newton-meter (N.m) units. The test-retest reliability of this method based on follow-up isokinetic testing done one week following the initial tests showed an intraclass correlation coefficient of 0.99.

#### 2.3.2. One Repetition Maximum

Total one-repetition maximum (1-RM) is the sum of the maximal weight a person can lift at one repetition for biceps curl, bench press, seated row, knee extension, knee flexion, and leg press [8, 10]. The test-retest reliability of this method based on follow-up 1-RM determinations one week following the initial tests showed an intraclass correlation coefficient of 0.96.

VO_2_  peak, a measure of aerobic fitness, was assessed during graded treadmill walking by indirect calorimetry (True Max 2400, ParvoMedics Salt Lake City, UT), as previously described [3, 11]. Briefly, the incremental test started at a speed determined, during a warm-period, to elicit ~70% of age-predicted HR_max⁡_, and the speed remained constant throughout the test, while the grade was increased by 2% every 2 minutes. The test continued until the subject could no longer exercise because of exhaustion or until other conditions, such as ECG changes or development of symptoms, made it unsafe to continue [12, 13].

#### 2.3.3. Body Weight

Body weight was measured in the morning after the subjects had fasted for 12 hours [8]. BMI was calculated as weight in kilograms/square of the height in meters (kg/m^2^).

#### 2.3.4. Biochemical Measurements

Blood samples were obtained in the morning after subjects fasted for at least 12 hours. Serum samples were extracted and stored at −80°C until analysis. Enzyme-linked immunosorbent assay kits were used to measure interleukin-6 (IL-6) (Quantikine, R&D Systems, Minneapolis, MN), soluble tumor necrosis factor receptor 1 (sTNFr1) (R&D, Minneapolis, MN, USA), and adiponectin (Quantikine, R&D Systems, Minneapolis, MN). Radioimmunoassay kits were used to measure serum estradiol (Ultra-sensitive estradiol DSL-4800; Diagnostic Systems Laboratories Inc., Webster, Tex), leptin (Leptin HL-81K; Linco Research Inc., St Charles, MO), and insulin-like growth factor 1 (IGF-1) (Diagnostic Products Group). High-sensitive C-reactive protein (Hs-CRP) was measured by immunoturbidimetric assay (Hitachi 917 analyzer), while serum total testosterone was measured by automated immunoassay (VITROS 5600). The CVs for these assays were <10%.

### 2.4. Statistical Analysis

Results are expressed as means ± SD. A *P* value of <0.05 was considered statistically significant. Normality for outcome variables was verified by Shapiro-Wilks test. Simple correlation analysis was performed to assess the individual associations between each hormone or adipokine with PPT, knee flexion, knee extension, total 1-RM, and VO_2_  peak followed by multiple regression analysis to determine the independent contribution of each hormone or adipokine to the above tests.

## 3. Results 

Our population consisted of 107 frail (PPT of ≤ 32) obese (BMI ≥ 30 kg/m^2^) and elderly (≥65 years old) males (*n* = 41) and females (*n* = 66). The baseline characteristics of these patients have been reported previously [8] and summarized in Table 1.

Table 2 showed significant positive correlations between the testosterone with the PPT, all measures of strength (knee flexion, knee extension, and total 1-RM), and aerobic fitness (VO_2_  peak), while no correlation was observed between estradiol and any of these measures. Significant positive correlation was observed between IGF-1 and measures of strength and aerobic fitness but not with modified PPT. Among the different adipokines, there was a negative correlation between the proinflammatory cytokine Hs-CRP with PPT, strength, and aerobic fitness (VO_2  _peak). The other pro-inflammatory cytokine IL-6 also correlated negatively with knee flexion and VO_2_  peak. The last pro-inflammatory cytokine tested sTNFr1 also correlated negatively with PPT, total 1-RM, and VO_2_  peak.

The adipokine leptin negatively correlated with PPT, all measures of strength, and VO_2_  peak. The adipokine adiponectin negatively correlated with total 1-RM.

Multiple regression analysis (Table 3) showed that, for PPT, sTNFr1 was the only independent predictor. Independent predictors for knee flexion included adiponectin, leptin and testosterone. Independent predictors for knee extension included: leptin and testosterone. Adiponectin, leptin and testosterone were independent predictors of total 1-RM while independent predictors of peak oxygen consumption (VO_2_  peak) included: IGF-1, IL-6, leptin and testosterone.

## 4. Discussion

Our results demonstrated that although there were correlations between gonadal and anabolic hormones and several adipokines with physical function, different measures of strength, and aerobic fitness (VO_2_  peak), sTNFr1 was the only independent predictor of overall physical performance (PPT), while testosterone seemed to be a consistent predictor of all measures of physical strength and fitness. Leptin and adiponectin also appeared to be independent determinants of the different measures of strength, while leptin additionally predicted VO_2_  peak. On the other hand, IGF-1 only independently predicted physical fitness, while estradiol appeared to have no role in physical function, strength, or fitness. Thus, our results suggest that circulating hormones and adipokines alone or in combination may contribute to frailty and physical disability in obese older adults.

Advancing age is associated with a decline in fat-free mass (FFM; primarily skeletal muscle) and function, known as sarcopenia [14]. Obesity is associated with higher absolute volume of FFM, but there is a disproportionately higher volume of fat mass relative to FFM resulting in relative muscle mass deficit exacerbating age-related muscle loss in the elderly obese [3]. Thus, obesity does not protect against sarcopenia and in fact it acts synergistically with sarcopenia (sarcopenic obesity) resulting in worst disability. Indeed, our group reported that 96% of obese elderly patients meet criteria for frailty [3].

Both obesity and aging have additive effects on chronic inflammation [15, 16] putting the elderly obese in a continuous state of heightened inflammation. Sarcopenic obesity is associated with increased levels of inflammatory cytokines [15] which have direct catabolic effects on skeletal muscle such as: TNF-*α* which suppresses muscle protein synthesis and interleukin-6 (IL-6) which inhibits the anabolic effects of IGF-1 [7]. Aside from adipose tissue production, skeletal muscles also express cytokines that have direct autocrine and paracrine effects within skeletal muscles [16]. Increased circulating concentrations of IL-6 are associated with lower muscle mass or strength and impaired mobility and in conjunction with low IGF-1 levels contribute synergistically to produce disability [17]. Our results showed that high levels of proinflammatory cytokines were independently associated with impaired strength and physical function and in fact, sTNFr1 was the only independent predictor of physical performance as assessed by the PPT score.

Our results also demonstrated a significant contribution of testosterone to physical strength and fitness, while estradiol appeared to have no role in physical function, strength, or fitness in our study population. Although part of decline in physical function and strength with aging is attributed to abnormalities in GH/IGF-1 production and signaling, our study showed that IGF-1 had very little role in these parameters in frail obese older men and women. Aging is associated with decrease in gonadal hormones in both sexes. In women, the drastic drop in estrogen levels with menopause is associated with reduction in muscle mass [18]. In men after age of 40, testosterone production gradually decreases at a rate of 1.6% per year for total and to 2-3% per year for bioavailable testosterone [19]. Because of the age-related increase in sex hormone binding globulin, the magnitude of the decrease in bioavailable testosterone in men is even greater than the decline in total testosterone levels [20]. This reduction in testosterone production in men parallels the (1) age-associated loss of muscle mass that leads to sarcopenia and impairment of function and (2) age-associated loss of bone mass that leads to osteopenia and fracture risk [21, 22].

In men, several randomized controlled trials (RCTs) have demonstrated thattestosterone significantly increases FFM (1.1 to 3.7 kg) and decreases fat mass (1.1 to 4.5 kg) [23–25]. In a review of seven trials, testosterone therapy was associated with a significantly greater increase in lean body mass (2.7 kg; 95% CI, 1.6–3.7) and a greater reduction in fat mass (−2.0 kg; 95% CI 3.1–0.8) than placebo [12]. The body weight change did not differ significantly between groups (−0.6 kg; 95% CI −2.0–0.8). In several studies, the increase in lean mass was accompanied by increase in muscle strength as assessed by hand grip strength, [25] maximum voluntary strength in a leg-press and chest-press exercise, [26] and isokinetic knee extension peak torque [27]. Furthermore, in a meta-analyses of 11 RCTs in elderly men, it was concluded that testosterone increased muscle strength particularly in the lower extremity (effect size: 0.63, 95% CI = 0.03–1.28) [28]. Moreover, testosterone replacement has been shown to improve physical function in healthy and mostly frail elderly men, as assessed by physical performance test [27], timed physical function test [25], aggregate locomotor function [27], and loaded stair-climbing [26].

On the other hand, although it is well-established that testosterone administration in postmenopausal women resulted in improvement in sexual function not only in surgically menopausal women but also in women with natural menopause [29–31], very few studies have investigated the effects of testosterone administration on body composition, muscle performance, and physical function in women. Huang et al. reported dose-related increases in lean body mass and improvement in strength [32] in postmenopausal women with low gender-adjusted testosterone suggesting the potential for testosterone to improve body composition and physical function in women. Their results were supported by data from another recent study showing that muscle protein synthesis in postmenopausal women is actually stimulated by testosterone and progesterone but not by estradiol [33].

Leptin and adiponectin share similarities in that both are produced by adipose tissues, have receptors in skeletal muscle, and have effects on muscle metabolism [34]. Leptin stimulates fatty acid oxidation in skeletal muscles and inhibits fat storage while promoting intramuscular lipolysis [35, 36]. Nevertheless, despite this positive effect of leptin on muscle metabolism, epidemiologic studies demonstrate a negative association between leptin with muscle mass and function [13, 37]. This inconsistency is believed to result from leptin resistance that develops with high-fat feeding and obesity [38, 39]. Similarly for adiponectin, aside from stimulating glucose utilization, it stimulates fatty acid oxidation and inhibits intramuscular fat lipid deposition [40, 41]. However, resistance to adiponectin in peripheral tissues similarly develops with high fat feeding and obesity [42] and may explain the inverse association found between adiponectin, with muscle strength and function [43, 44]. Thus, these adipokines may be useful as biomarkers for frailty.

Our study has limitations. It is cross-sectional study and therefore does not provide a direct evidence of the actual physiologic role of each factor on physical function, strength, and frailty. In addition, our sample size was relatively small which thus needs confirmation in a larger population of obese older adults.

In summary, our results indicate that circulating testosterone levels and adipokines alone or in combination influence physical function, strength, and aerobic fitness in the frail obese older adults. Our group previously demonstrated that lifestyle intervention by diet and exercise was safe and improves physical function and ameliorates frailty in this population [8]. However, we also showed that although weight loss-associated muscle and bone loss can be attenuated by exercise, it did not totally prevent these complications. Given testosterone's effect on increasing bone density, muscle mass and strength, whether adding testosterone to lifestyle therapy, would be able to prevent muscle and bone loss from weight loss, while at the same time improving strength needs to be examined in future studies.

## Figures and Tables

**Table 1:** Clinical and biochemical characteristics of study participants.

Age (years)	69.4 ± 4.1
BMI (kg/m^2^)	37.0 ± 4.9
Weight (kg)	116.7 ± 12.9
Testosterone (ng/dL)	119.3 ± 151.8
Estradiol (pg/mL)	52.5 ± 22.6
Adiponectin (ng/mL)	25.0 ± 15.3
Leptin (*μ*g/mL)	36.6 ± 22.6
IL-6 (pg/mL)	2.3 ± 2.5
Hs-CRP (mg/L)	4.1 ± 4.5
sTNFr1 (pg/mL)	167 ± 43.4
PPT (points)	27.6 ± 3.3
Knee flexion (Nm)	47.7 ± 16.4
Knee extension (Nm)	71.2 ± 25.5
Total 1-RM (lb.)	542.7 ± 194.0
VO_2_ peak (mL/kg/min)	17.2 ± 3.3

BMI: Body mass index, IL-6: interleukin-6, Hs-CRP: high sensitivity C-reactive protein, sTNFr-1: soluble tumor necrosis factor receptor-1, PPT: physical performance test, Total 1-RM: total 1-repetition max, VO_2_ peak: peak oxygen consumption, and Nm: Newton meter.

**Table 2:** Simple correlation analysis between physical function, strength, and aerobic fitness with different hormones and adipokines.

	Testosterone	Estradiol	IGF-1	IL-6	sTNFr1	Hs-CRP	Leptin	Adiponectin
Modified PPT	0.23*	−0.03	0.10	−0.11	−0.30**	−0.22*	−0.22*	−0.01
Knee flexion	0.49**	0.14	0.26*	−0.25*	−0.15	−0.22*	−0.53**	−0.11
Knee extension	0.51**	0.18	0.26**	−0.16	−0.14	−0.26**	−0.44**	−0.10
Total 1-RM	0.66**	0.14	0.23*	−0.13	−0.20*	−0.26**	−0.52**	−0.22*
VO_2_ peak	0.56**	0.04	0.20*	−0.28**	−0.23*	−0.28**	−0.61**	−0.09

Data are mean ± SD. *P < 0.05; **P < 0.01. VO_2_ peak: peak oxygen consumption, Nm: Newton meter, BMI: body mass index, IL-6: interleukin-6, Hs-CRP: high sensitivity C-reactive protein, sTNFr-1: soluble tumor necrosis factor receptor-1, PPT: modified physical performance test, and Total 1-RM: total 1-repetition max.

**Table 3:** Final model of the hormonal and adipokine predictors of physical performance test, strength measures, and aerobic fitness (VO_2_ peak) in frail obese older adults.

	*R* ^2^ (%)	Beta estimate	SE	*P*
PPT	10			
sTNFr1		−0.025	0.008	0.003
Knee flexion	37.9			
Adiponectin		−0.37	0.16	0.02
Leptin		−0.29	0.09	0.002
Testosterone		0.034	0.013	0.01
Knee extension	27.4			
Leptin		−0.28	0.13	0.04
Testosterone		0.06	0.02	0.003
Total 1-RM	54.3			
Adiponectin		−3.22	1.07	0.004
Leptin		−2.23	0.81	0.006
Testosterone		0.71	0.12	<0.001
VO_2_ peak	54.3			
IGF-1		0.02	0.01	0.02
IL-6		−0.31	0.11	0.009
Leptin		−0.05	0.01	<0.001
Testosterone		0.007	0.002	0.001

Variables entered in the model: sTNFr1, adiponectin, leptin, testosterone, estradiol, IGF-1, IL-6, leptin, age, and sex. PPT: modified physical performance test, Total 1-RM: total 1-repetition max, VO_2_ peak: peak oxygen consumption, sTNFr1: soluble tumor necrosis factor receptor-1, IGF-1: insulin growth factor-1, and IL-6: interleukin-6.
